# Editorial: Molecular Nanomachines of the Presynaptic Terminal, Volume II

**DOI:** 10.3389/fnsyn.2022.941339

**Published:** 2022-06-07

**Authors:** Lucia Tabares, Silvio O. Rizzoli

**Affiliations:** ^1^Department of Medical Physiology, School of Medicine, University of Seville, Seville, Spain; ^2^Instituts für Neuro- und Sinnesphysiologie Zentrum Physiologie und Pathophysiologie, Universitätsmedizin Göttingen Georg-August-Universität, Göttingen, Germany

**Keywords:** synapse, exocytosis, endocytosis, neurons, nanomachines

The synapse has been designed and refined during animal evolution for two main processes: (1) to translate and transmit information with exquisite spatio-temporal precision, and (2) to prevent the spread of unspecific signals. This resulted in a highly specialized arrangement of molecules, especially on the presynaptic side, to enable the synapse to respond to these pressures. For example, the exocytosis machinery needs to be exquisitely sensitive to meaningful signals and respond precisely to different stimulation patterns. The endocytosis machinery needs to balance exocytosis to prevent changes in synapse shape and volume, but also needs precision in the selective removal of vesicular material, rather than random uptake of membrane components.

Multiple machines (nanomachines) need to be coordinated to organize this activity and keep it in tune with the long-term needs of the neuron and the synapse. Proteins and vesicles become “used” and need to be eventually degraded, either locally or in lysosomes located elsewhere, which poses significant logistic challenges. Some of these challenges need to be solved within the synaptic vesicle cluster, while others require the re-organization of the synaptic release sites. Moreover, the synapse as a whole needs to maintain its organization and modulate its shape and size dynamically in relation to the activity and plasticity needs of the neuronal network. Actin is among the most important elements that modulate these aspects, being responsible for both vesicle dynamics and maintaining the plasma membrane shape (*via* cortical actin). In addition, the extracellular matrix also participates in the maintenance of synapse shape and connectivity.

Our Research Topic presents a timely and thorough view of many of these synaptic elements, starting at the level of individual proteins, proceeding up to complex plasticity and re-shaping mechanisms. Two of the most abundant proteins of the synapse, synaptobrevin (VAMP2) and synaptophysin, are revised by White and Stowell, who discuss their organization in the synapse, especially in relation to how these molecules impact the traffic of vesicles to the presynaptic membrane and the formation of the fusion complex. The clear conclusion is that these molecules form complexes involved in multiple functions, fully deserving the term “nanomachines.”

One long-standing issue has been that such molecules are typically analyzed in static conditions, while their functions are most evident during phases of activity. To solve this problem, Jackson et al. present here the combination of live imaging with structural imaging methods at the super-resolution level. This type of approach enables a thorough functional view of the synaptic complex and should enable many discoveries in the future. At the same time, imaging synaptic proteins can be combined with the direct investigation of other elements, such as the neurotransmitters themselves. For analyzing these components, Lork et al. present chemical imaging methods that can be implemented both in fixed and live cells.

Imaging methods have long served the synaptic exocytosis field and have led, more than a decade ago, to the conclusion that not every exocytosis event is the same as any other. Synaptic vesicles and active zones are heterogenous and specialized for different release flavors, even within the same synapse, as explored by Guzikowski and Kavalali. Nevertheless, all fused vesicles need to be eventually retrieved and re-formed. This process is complex, and also connects to the degradation system in complex fashions involving the endo-lysosomal molecules. The involvement of these molecules in the synaptic vesicle recycling system is analyzed by Ivanova and Cousin. Importantly, as implied above, protein degradation does serve not only the synaptic vesicle cycle but also other synaptic processes. Here the evidence on the involvement of different presynaptic structures with membrane-based protein-degradation pathways is reviewed by Gundelfinger et al., concluding that synaptic vesicles, their endocytic machinery, and the main components of the release sites are strongly connected to degradation pathways.

Synaptic proteins and vesicles are often connected by actin, which participates in many of the dynamic processes of the synapse. Actin is involved in almost all forms of endocytosis, in the vesicle replenishment to the readily releasable pool, the expansion of the fusion pore, and even in vesicular content release, as described by Wu and Chan.

While synaptic vesicles have been known to undergo exo- and endocytose for decades, this is less known for other synaptic components. Surprisingly, extracellular matrix molecules do it as well, in a fashion that relates to synaptic vesicle dynamics (Dankovich and Rizzoli), which presumably represents a new form of plasticity involved in re-shaping the synaptic release sites and adjusting them to the structure of the post-synapse.

The release sites themselves are also complex and dynamic and are functionally linked to and regulated by mitochondria, as Lopez-Manzaneda et al. demonstrate. The mitochondria act mainly by buffering the local Ca^2+^ concentrations, thereby regulating the fusion of the synaptic vesicles during activity bursts (rendering it synchronous or asynchronous). This finding enhances our current understanding of the regulation of the release site function since most previous works have concentrated on Ca^2+^ dynamics related to the entry of ions through membrane channels that are located at the release sites and were therefore thought to be more likely to affect vesicle release than the more distantly located mitochondria. While it is not yet clear whether the mitochondria-based regulation also involves modulation of the release site architecture, Holderith et al. show here that such regulation, including the selective placement and organization of specific proteins, is more common than previously thought. This type of regulation may even be a strong component of presynaptic plasticity since the release sites, along with the synaptic vesicles, are among the primary targets for plasticity-related alterations, as discussed by Shahoha et al.

The works in this topic provide, therefore, a glimpse into the complex organization of the presynapse, from single molecules to plasticity mechanisms. In comparison to the similarly titled topic of 6 years ago, we can conclude that, while the number of targets and nanomachines increased strongly, so did our knowledge of the intricate synaptic dynamics. The major progress in synapse-describing tools that has ensued over the last years also offers the hope that even more quantitative works can be performed in the future, providing an even more precise understanding of the function of this essential brain component.

## Author Contributions

All authors listed have made a substantial, direct, and intellectual contribution to the work and approved it for publication.

## Conflict of Interest

The authors declare that the research was conducted in the absence of any commercial or financial relationships that could be construed as a potential conflict of interest.

## Publisher's Note

All claims expressed in this article are solely those of the authors and do not necessarily represent those of their affiliated organizations, or those of the publisher, the editors and the reviewers. Any product that may be evaluated in this article, or claim that may be made by its manufacturer, is not guaranteed or endorsed by the publisher.

